# Sulphur and Malaria (Extract)

**Published:** 1882-12

**Authors:** 


### SULPHUR AND MALARIA.

At a recent meeting of the Paris Academy, M. d'Abbadie called attention to some facts regarding marsh fever. Some African elephant hunters from plateaus with comparatively cool climate, brave the hottest and most deleterious Ethiopian regions with impunity, which they attribute to their habit of daily fumigation of the naked body with sulphur. It is interesting to know whether sulphurous emanations, received involuntarily, have a like effect. From inquiries made by M. Fouque, it appears that in Sicily, while most of the sulphur mines are in high districts and free from malaria, a few are at a low level, where intermittent fever prevails. In the latter districts, while the population of the neighboring villages is attacked by fever in the proportion of ninety per cent., the workmen in the sulphur mines suffer much less, not more than eight or nine per cent. being attacked. Some other facts tending to show the anti-malarial influence of sulphur are given.—*Medical Record, Nov. 4th.*

